# miR-593 inhibits proliferation and invasion and promotes apoptosis in non-small cell lung cancer cells by targeting SLUG-associated signaling pathways

**DOI:** 10.3892/mmr.2021.12555

**Published:** 2021-12-06

**Authors:** Fang Wei, Mofei Wang, Zhen Li, Yong Wang, Yong Zhou

Mol Med Rep 20: 5172-5182, 2019; DOI: 10.3892/mmr.2019.10776

Subsequently to the publication of this paper, an interested reader drew to the authors’ attention that Fig. 5D on p. 5180 appeared to contain a pair of data panels with overlapping data comparing between the H1299/NC and H1299/Mimic experiments, such that these data panels may have been derived from the same original source.

The authors have consulted their original data, and realized that one of the images was inadvertently selected inappropriately for the figure. Nevertheless, they were able to present all the original data to the Editorial Office, and the repeated experiments revealed the same trends in terms of significant differences in cell migration and invasion. The corrected version of Fig. 5, showing all the correct data for Fig. 5D, is shown on the next page. Note that the errors in Fig. 5 did not significantly affect the results or the conclusions reported in this paper, and all the authors agree to this Corrigendum. The authors are grateful to the Editor of *Molecular Medicine Reports* for allowing them the opportunity to publish this corrigendum, and apologize to the readership for any inconvenience caused.

## Figures and Tables

**Figure 5.**
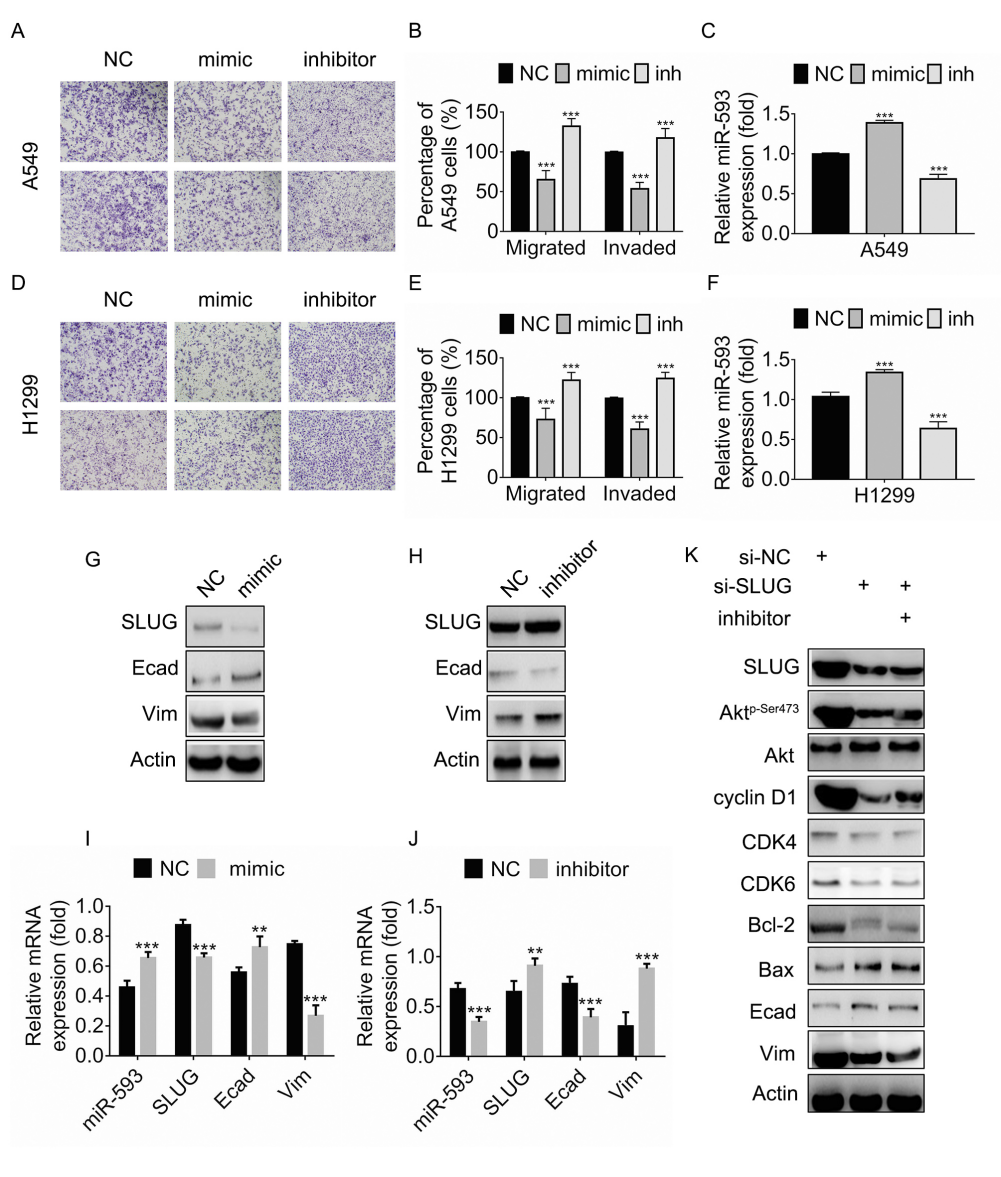
miR-593 suppresses cellular migration and invasion via downregulation of SLUG expression. (A) Migration assays (top panel) and invasion assays (bottom panel) of A549 cells. Cells were treated with NC, miR-593 mimic or miR-593 inhibitor for 24 h. Migration assay (without Matrigel) and Matrigel invasion assay were performed to evaluate the changes in cellular motility. Representative photomicrographs of Transwell results were captured under an ×100 magnification. (B) Percentage of migrated or invaded A549 cells. Cells in 5 visual fields at ×400 magnification were counted in each group, and the percentage was calculated in comparison with the NC group. (C) Relative miR-593 expression was determined by RT-qPCR. (D) Migration assays (top panel) and invasion assays (bottom panel) of NCI-H1299 cells. Cells were treated with NC, miR-593 mimic or miR-593 inhibitor for 24 h. Migration assay (without Matrigel) and Matrigel invasion assay was performed to investigate the changes in cellular motility. Representative photomicrographs of the results were obtained under an ×100 magnification. (E) Percentage of migrated or invaded NCI-H1299 cells. Cells in 5 visual fields at ×400 magnification were counted in each group, and the percentage was calculated in comparison with the NC group. (F) Relative miR-593 expression was evaluated by RT-qPCR. (G) SLUG, E-cadherin and vimentin expression in A549 cells treated with NC or miR-593 mimic, were detected by western blotting. (H) The levels of the indicated proteins were examined in cells treated with NC or miR-593 inhibitor. (I) A549 cells were transfected with NC alone or miR-593 mimic for 24 h, and the relative mRNA expression of miR-593, SLUG, E-cadherin and vimentin was then determined by RT-qPCR. (J) Cells were transfected with NC alone or miR-593 inhibitor for 24 h, and the relative mRNA expression of miR-593, SLUG, E-cadherin and vimentin was then determined by RT-qPCR. (K) A549 cells were treated with siRNA NC, SLUG siRNA or a combination of SLUG siRNA and miR-593 inhibitor for 24 h. Western blotting was carried out upon treatment to verify the changes in the expression levels of the indicated proteins. The results represent the mean ± standard deviation of 3 independent experiments. **P<0.01, ***P<0.001. miRNA, microRNA; SLUG, zinc finger protein SNAI2; NC, negative control; mimic, miR-593 mimic; inh, miR-593 inhibitor; Akt, protein kinase B; CDK4, cyclin-dependent kinase 4; CDK6, cyclin-dependent kinase 6; Bcl-2, apoptosis regulator Bcl-2; Bax, apoptosis regulator BAX; Ecad, E-cadherin; Vim, vimentin; si-NC, siRNA negative control; si-SLUG, SLUG siRNA; siRNA, small interfering RNA; RT-qPCR, reverse transcription-quantitative polymerase chain reaction; mRNA, messenger RNA; H1299, NCI-1299.

